# Solitary Hydatid Cysts of the Body: Analysis of 1303 Cases

**DOI:** 10.5152/eurasianjmed.2025.251086

**Published:** 2025-11-14

**Authors:** Yener Aydın, Ali Bilal Ulaş, Suat Eren, Gürkan Öztürk, Kamber Kaşali, Binali Fırıncı, Mehmet Kürşat Karadağ, Eyüp Şenocak, Sevilay Özmen, Yılmaz Aksoy

**Affiliations:** 1 Department of Thoracic Surgery, Atatürk University Faculty of Medicine, Erzurum, Türkiye; 2 Department of Radiology, Atatürk University Faculty of Medicine, Erzurum, Türkiye; 3 Department of General Surgery, Atatürk University Faculty of Medicine, Erzurum, Türkiye; 4 Department of Biostatistics, Atatürk University Faculty of Medicine, Erzurum, Türkiye; 5 Department of Pediatric Surgery, Atatürk University Faculty of Medicine, Erzurum, Türkiye; 6 Department of Neurosurgery, Atatürk University Faculty of Medicine, Erzurum, Türkiye; 7 Department of Orthopedics and Traumatology, Atatürk University Faculty of Medicine, Erzurum, Türkiye; 8 Department of Cardiovascular Surgery, Atatürk University Faculty of Medicine, Erzurum, Türkiye; 9 Department of Urology, Atatürk University Faculty of Medicine, Erzurum, Türkiye

**Keywords:** Hydatid disease, kidney, liver, lung, solitary, spleen, surgery

## Abstract

**Background:**

This study aimed to investigate solitary hydatid cysts occurring in various anatomical locations.

**Methods:**

A retrospective single-center analysis was conducted on 1303 consecutive cases of solitary hydatid cysts identified across different body regions between 2015 and 2024.

**Results:**

Solitary hydatid cysts accounted for 67.7% (1303/1926) of all treated cases. Among the 1303 patients, 609 (46.7%) were male and 694 (53.3%) were female. The mean age was 35.3 ± 20.4 years, with a range from 2 to 87 years. Among pediatric cases, 153 (43.6%) were female and 198 (56.4%) were male, whereas among adults, 541 (56.8%) were female and 411 (43.2%) were male (*P* < .01). Cyst localization was predominantly hepatic (969 cases, 74.3%), followed by pulmonary (250 cases, 19.1%). Other sites included the spleen (21 cases, 1.6%), muscle tissue (14 cases, 1.1%), kidneys (11 cases, 0.8%), bones (10 cases, 0.8%), intracranial areas (8 cases, 0.6%), cardiac regions (6 cases, 0.5%), mediastinum (3 cases, 0.2%), pelvic space (3 cases, 0.2%), subcutaneous and soft tissue (3 cases, 0.2%), omentum (3 cases, 0.2%), diaphragm (1 case, 0.1%), and pancreas (1 case, 0.1%). The mean diameter of hydatid cysts was 72.5 ± 33.2 mm (range: 10-230 mm). Surgical intervention was the primary treatment across all cases; however, medical therapy was more frequently selected for cysts located in the liver and spleen than in other anatomical regions.

**Conclusion:**

Solitary hydatid cysts typically affect the liver and lungs but may arise in any location. Surgery remains the primary treatment across all sites.

**Main Points**

- Solitary hydatid cysts most commonly occur in the liver and lungs, but may arise in any anatomical location.
- Pediatric patients are more frequently male, whereas adult patients are predominantly female.
- Diagnosis of solitary cysts may be more challenging compared to multiple cyst presentations.
- Treatment may be delayed, particularly in solitary cysts located outside the lungs and liver.

## Introduction

Hydatid disease is transmitted to humans via contact with dogs or ingestion of unwashed vegetables, fruits, or contaminated water. Once ingested, the embryos released from the eggs attach to the intestinal wall—especially in the duodenum—where bile salts dissolve the embryonic shell. The embryos penetrate the jejunal and ileal walls, entering the portal vein or periduodenal and perigastric lymphatics, typically reaching the liver. If not retained in the liver, they may travel to the lungs or disseminate through systemic circulation, potentially lodging in any organ and causing disease.^1^^,^^2^ Hydatid cysts may present as solitary lesions, multiple cysts within a single organ, or concurrent involvement of multiple organs.^3^^,^^4^ This study investigates solitary hydatid cysts occurring in diverse anatomical locations.

## Materials and Methods

This retrospective study was conducted at Atatürk University Medical Faculty following approval from the Institutional Review Board and Ethics Committee (Approval no: B.30.2.ATA.0.01.00/581; Date: September 27, 2024), in accordance with the Declaration of Helsinki.

This single-center retrospective study analyzed 1303 solitary hydatid cyst cases treated between 2015 and 2024, reviewing patient demographics, cyst characteristics, clinical presentations, and treatment approaches.

### Statistical Analysis

Statistical analyses were conducted using IBM SPSS 20 (IBM SPSS Corp.; Armonk, NY, USA). Descriptive data were presented as mean, SD, median, minimum, maximum, percentage, and count. For categorical variables, the Pearson chi-square test was applied when expected values exceeded 5, the Yates correction for values between 3 and 5, and Fisher’s exact test for values below 3. A *P*-value < .05 was considered statistically significant.

## Results

**Figure 1.**
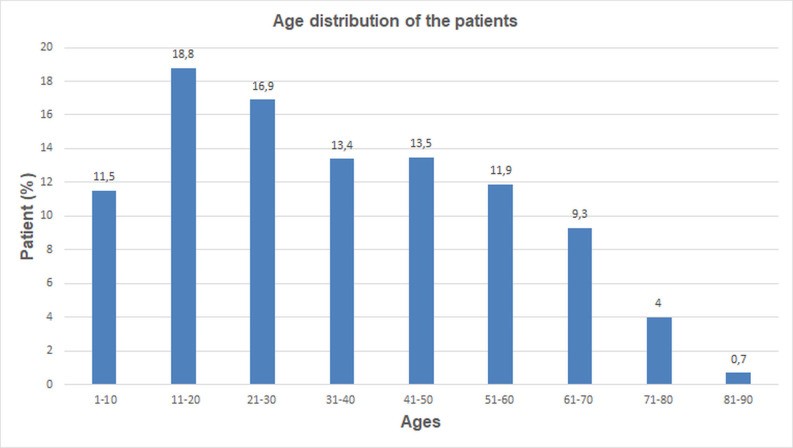
Age distribution of patients.

**Figure 2.**
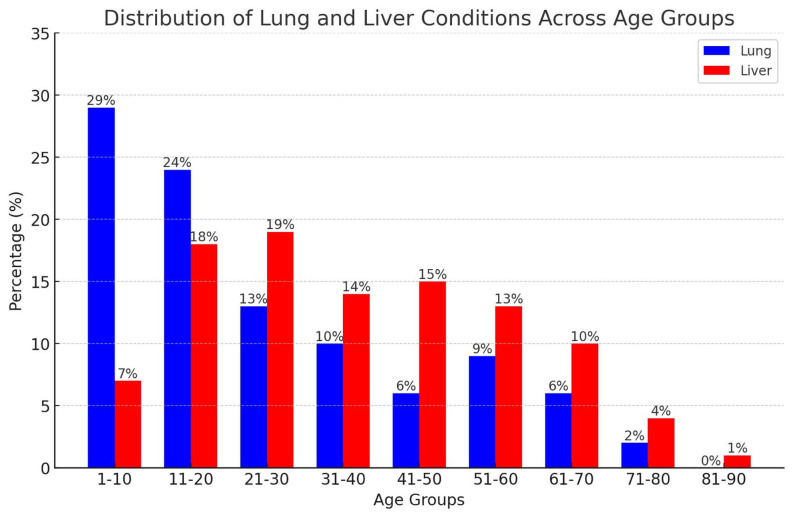
Distribution of liver and lung hydatid cysts among age groups.

Solitary hydatid cysts comprised 67.7% (1303/1926) of all treated cases. Most patients resided in rural areas (1101; 84.5%), while 202 (15.5%) lived in urban settings (*P* < .01). The cohort included 609 males (46.7%) and 694 females (53.3%), with a mean age of 35.3 ± 20.4 years (range: 2-87). A total of 351 patients (26.9%) were children (≤18 years), while 952 (73.1%) were adults (>18 years) (Figure 1). Among children, 198 were male (56.4%) and 153 female (43.6%); among adults, 541 were female (56.8%) and 411 male (43.2%) (*P* < .01). Lung involvement was highest in the 1-10 age group (29%) and declined with age, whereas liver involvement peaked in the 21-30 group (19%) and remained more prevalent in middle age (*P* < .01) (Figure 2).

**Figure 3.**
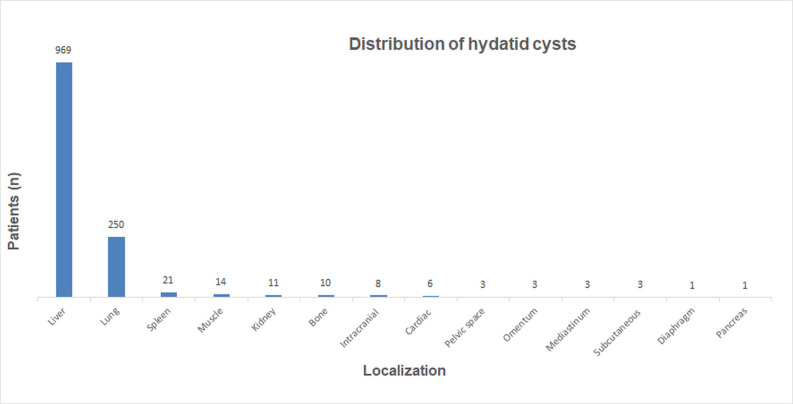
Localization distribution of hydatid cysts.

Cyst localization was primarily hepatic (969 cases, 74.3%), followed by pulmonary (250, 19.1%). Less frequent sites included the spleen (21, 1.6%), muscle tissue (14, 1.1%), kidneys (11, 0.8%), bones (10, 0.8%), intracranial areas (8, 0.6%), heart (6, 0.5%), mediastinum, pelvic space, subcutaneous tissue, omentum (3 cases each, 0.2%), and diaphragm and pancreas (1 case each, 0.1%) (Figure 3). The mean cyst diameter was 72.5 ± 33.2 mm (range: 10-230 mm).

Presenting symptoms varied by cyst location. In hepatic cases, abdominal pain was most common (655; 67.6%), followed by nausea and vomiting (105; 10.8%) and chest pain (72; 7.4%). Pulmonary cases most frequently presented with cough (132; 52.8%), followed by chest pain (99; 39.6%) and dyspnea (45; 18%) (Table 1).

The World Health Organization Informal Working Group on Echinococcosis (WHO-IWGE) classification for 969 hepatic hydatid cysts was CE1 in 327 cases (33.8%), CE2 in 171 (17.6%), CE3 in 190 (19.6%), CE4 in 157 (12.2%), and CE5 in 124 (12.8%). The right lobe was affected in 696 cases (71.8%), the left in 238 (24.6%), and both lobes in 35 (3.6%).

Treatment methods for liver hydatid cysts included surgery in 363 cases (37.5%), medical therapy with the “watch and wait” approach in 350 cases (36.1%), percutaneous therapy in 236 cases (24.3%), and a combination of surgery and percutaneous therapy in 20 cases (2.1%).

Pulmonary hydatid cysts were identified in 250 individuals, with 120 intact (48%) and 130 ruptured (52%). Right-sided pulmonary involvement was observed in 143 individuals (57.2%), exceeding left-sided localization noted in 107 (42.8%). The right lower lobe was most frequently affected (91; 36.4%), followed by the left lower lobe (73; 29.2%). Surgical treatment was administered in 241 cases (96.4%), while medical therapy alone was used in 9 (3.6%).

Spleen involvement was noted in 21 individuals. Surgical intervention was performed in 9 cases (42.9%), medical therapy with a “watch and wait” approach was used in 7 cases (33.3%), and percutaneous puncture-aspiration-injection-reaspiration (PAIR) was applied in 5 cases (23.8%).

Muscle involvement was documented in 14 individuals, predominantly affecting the thigh and lower extremities. Surgical treatment was applied in 11 (78.6%), and PAIR in 3 (21.4%).

Kidney involvement was observed in 11 cases, with 6 (54.5%) in the right kidney and 5 (45.5%) in the left. Surgical intervention was performed in 9 (81.8%), while medical therapy was administered in 2 (18.2%).

Osseous involvement was documented in 10 individuals: vertebrae (n = 4), femur (n = 1), rib (n = 1), iliac wing (n = 1), sacrum (n = 1), ulna (n = 1), and clavicle (n = 1). Surgical treatment was administered in 8 cases, while medical therapy was reserved for sacral and iliac lesions (n = 2). Diagnosis was confirmed histopathologically via biopsy.

Intracranial involvement was identified in 8 individuals, all children or young adults (mean age: 14.3 years). Lesions were located in the frontal (n = 2), temporal (n = 2), frontoparietal (n = 1), frontotemporal (n = 1), parieto-occipital (n = 1), and parietal (n = 1) regions. All patients underwent surgical treatment.

Cardiac involvement was noted in 6 individuals, affecting the left ventricle (n = 3), right ventricle (n = 2), and interventricular septum (n = 1). Surgical intervention was performed in 5 patients, while 1 received medical therapy after declining surgery.

Uncommon hydatid cyst localizations—mediastinum, pelvic cavity, subcutaneous tissue, omentum, diaphragm, and pancreas—were all managed surgically (Table 2). These accounted for a minor subset of the cohort and included both pediatric and adult patients. Notably, a mediastinal cyst was identified in a 6-year-old child, while the remaining cases were reported in adults.

One patient with hepatic hydatid cysts developed postoperative multi-organ failure following surgery, resulting in death. Another patient who declined surgical treatment died 2 years later due to disease-related complications.

## Discussion

Hydatid cyst remains a notable public health issue, especially in rural regions with widespread livestock farming.^5^^-^^7^ The liver is the most frequently affected organ (70%-80%), followed by the lungs (10%-30%), while other sites, including the spleen, kidneys, heart, brain, and bones, account for approximately 10% of cases.^6^^,^^8^ Previous studies indicate that 80%-90% of hydatid disease cases affect a single organ, and 70%-80% present with a solitary cyst.^5^^-^^9^ In this study, 84.5% of cases originated from rural areas. Solitary cysts constituted 67.7% of treated hydatid cysts, most frequently located in the liver (74.3%) and lungs (19.1%), with 6.6% in other regions.

Cyst distribution varies by gender, age group, and geographic origin.^10^ The lungs are reported as the most common site of cyst development in children.^2^^,^^3^^,^^8^ Similarly, 50%-75% of intracranial hydatid cysts occur in pediatric and adolescent age groups.^11^^,^^12^ Khazaei et al^7^ reported that approximately 40% of cases involved individuals aged 21-40 years. Women accounted for approximately 55% of cases, while men represented 45%.^5^^,^^7^ This gender distribution may be associated with domestic activities involving exposure to infected dogs, soil, and vegetables; earlier detection through routine abdominal ultrasounds; and hormonal factors such as elevated estrogen levels.^13^ In this study, 46.3% of cases involved men and 53.3% women. Among children, 43.6% were female and 56.4% male; among adults, 56.8% were female and 43.2% male. Lung involvement was highest in the first decade, decreasing with age, while liver involvement peaked in the third decade and remained more common in middle-aged individuals. Intracranial involvement was observed exclusively in children and young adults.

According to WHO-IWGE, hydatid cysts are categorized into 5 types within 3 groups: CE1 and CE2 indicate active infection, CE3 denotes the transitional stage, and CE4 and CE5 correspond to inactive cysts.^1^ Liver cysts predominantly involve the right lobe (80%).^2^^,11^ Pulmonary cysts are mainly located in the lower lobes (60%), with reported rupture rates ranging from 50% to 90%.^8^^,^^10^^,^^11^ In this study, 33.8% of hepatic cysts were classified as CE1, 17.6% as CE2, 19.6% as CE3, 12.2% as CE4, and 12.8% as CE5. The right hepatic lobe was affected in 71.8% of cases, the left lobe in 24.6%, and both lobes in 3.6%. This data showed that 48% of pulmonary cysts were intact and 52% were ruptured. Pulmonary localization was observed in the right lung in 57.2% of cases and in the left lung in 42.8%, with the right lower lobe (36.4%) and left lower lobe (29.2%) being the most affected sites.

The incidence of primary and isolated cysts in rare locations is low. Following the liver and lungs, the spleen is the third most affected organ, with primary splenic cysts occurring at a rate of 0.2% to 2%.^11^^,^^14^^,15^ Renal involvement occurs in 2%-3% of cases, while primary musculoskeletal involvement accounts for less than 1%.^14^^-^^16^ The most frequent muscular sites are the thigh, followed by the paravertebral region.^16^ Bone involvement is seen in 0.5%-2% of cases, most frequently in the spine, followed by the pelvis, long bones, skull, and ribs.^2^^,^^11^ Intracranial cysts constitute 1% of cases, with the parietal region being the most common site, followed by the frontal, temporal, and occipital regions.^2^^,^^12^ Cardiac hydatid cysts occur sporadically, representing 0.02%-2% of cases, most frequently in the left ventricle.^2^^,^^11^^,^^17^ Additional localizations have been reported in case studies, involving other anatomical regions.^18^^-^^22^ In this study, unusual cyst localizations included the spleen (1.6%), muscle tissue (1.1%), kidney (0.8%), bone (0.8%), intracranial region (0.6%), heart (0.5%), mediastinum (0.2%), pelvic space (0.2%), subcutaneous and soft tissue (0.2%), omentum (0.2%), diaphragm (0.1%), and pancreas (0.1%).

Clinical manifestations of hydatid cysts vary by localization and may be asymptomatic. Liver cysts commonly present with abdominal pain and anorexia, while intra-abdominal cysts exhibit similar symptoms. Pulmonary cysts typically cause chronic cough, dyspnea, pleuritic chest pain, and hemoptysis.^23^^-^^25^ Renal cysts may cause low back or flank pain, hematuria, and hypertension.^14^ Intracranial cysts typically present with headache, nausea, vomiting, and elevated intracranial pressure.^25^ Muscle involvement usually manifests as a painless, slow-growing mass beneath normal skin.^16^ Cardiac cysts may present with chest pain, dyspnea, hemoptysis, and anaphylaxis.^2^ In this study, liver-localized cases most commonly manifested as abdominal pain (67.6%), nausea and vomiting (10.8%), and chest pain (7.4%). For pulmonary cysts, the leading symptoms were cough (52.8%), chest pain (39.6%), and dyspnea (18%).

Treatment strategies depend on cyst stage, size, symptoms, complications, and localization. Available options include medical therapy, surgical resection, PAIR, and catheterization.^2^^,^^24^ Partial and total cystectomy have traditionally represented the most common and definitive surgical interventions for hydatid cysts.^9^^,^^25^

Surgical management of liver cysts includes conservative and radical approaches. Radical procedures such as total cystectomy and hepatic resection may result in notable morbidity despite the generally benign nature of the disease. Consequently, conservative methods like partial cystectomy are more frequently employed.^25^ Surgical intervention is preferred for large CE2–CE3b cysts containing multiple daughter cysts, superficially located cysts with rupture risk, and cysts exerting pressure on adjacent vital organs.^22^^,^^23^ Anthelmintic therapy is indicated for uncomplicated CE1 and CE3a liver cysts smaller than 5 cm, multiple liver cysts, or inoperable cases with multi-organ involvement. It may also be used as a neoadjuvant treatment prior to percutaneous or surgical intervention to inactivate the cyst, reduce wall tension, and minimize the risk of intraoperative rupture. Albendazole is the preferred anthelmintic agent due to its higher bioavailability and superior intestinal absorption.^1^^,^^2^ Percutaneous techniques include PAIR, standard catheterization, and the modified catheterization technique (MoCat). Puncture-aspiration-injection-reaspiration therapy entails cystic fluid aspiration under ultrasound or computed tomography guidance and is effectively applied to single-compartment cysts such as CE1 and CE3a, smaller than 10 cm.^24^^,^^25^ The “watch and wait” strategy is applicable to inactive CE4 and CE5 liver cysts, with follow-up conducted through periodic ultrasound, without pharmacological or surgical intervention.^9^^,^^25^ In this study, 37.5% of liver cysts were managed surgically, 36.1% received medical therapy combined with the “watch and wait” approach, 24.3% were treated solely by percutaneous methods, and 2.1% underwent both percutaneous and surgical interventions.

For pulmonary hydatid cysts, surgical assessment is the initial step upon detection. Unlike hepatic cysts, PAIR is contraindicated. Albendazole is not recommended prior to surgery for intact cysts due to rupture risk. The most frequently used surgical techniques are cystotomy and capitonnage, which aim to preserve lung parenchyma.^26^^,^^27^ In this study, 96.4% of cases were treated surgically, while 3.6% received medical therapy alone.

For splenic hydatid cysts, total or partial splenectomy, cyst enucleation, and unroofing with omentoplasty are applicable. Puncture-aspiration-injection-reaspiration may be considered in patients with poor overall condition, while medical therapy serves as a palliative option for inoperable cases.^15^ Surgery is the preferred approach for renal hydatid cysts. Superficial cysts with limited parenchymal involvement may be treated with deroofing or pericystectomy, while partial or total nephrectomy is indicated for cases with extensive renal involvement.^14^ Intracranial cysts necessitate surgical management in all cases, with techniques such as the Dowling, Arana-Iniguez, and pericystic hydraulic methods applied across study groups.^12^ Due to the absence of effective medical therapy for cardiac involvement, surgical intervention is required.^17^ For cysts in other anatomical regions, surgery likewise constitutes the principal treatment modality.^19^ In this study, surgery was the primary treatment modality for unusual localizations, with surgical rates of 42.9% for the spleen, 78.6% for muscle tissue, 81.8% for kidneys, 80% for bones, and 83.3% for cardiac involvement. All intracranial and other rare localizations were treated surgically.

This study has several limitations. First, it is retrospective. Second, clinical and radiological comparisons could not be conducted due to variations in case characteristics and localizations. Third, in liver and spleen cysts treated with medical therapy or a “watch and wait” approach, the distinction between the 2 was unclear and therefore combined. Fourth, PAIR and catheter techniques were grouped under percutaneous therapy without individual comparison. Finally, regional differences in postoperative and long-term outcomes limited comprehensive evaluation.

As a result, solitary hydatid cysts may occur at various anatomical sites, most commonly in the liver and lungs. They can present at any age, with peak incidence observed in the second decade of life. While surgical intervention remains the primary treatment modality across all localizations, percutaneous and medical therapies, as well as the “watch and wait” strategy, play important roles in the management of liver and splenic cysts.

## Figures and Tables

**Table 1.** Distribution of Symptoms

Localization	Symptoms
Liver (n = 969)	Abdominal pain (655), nausea and vomiting (105), chest pain (72), weakness and anorexia (68), swelling in the abdominal wall (40), dyspnea (25), bloating sensation (15), headache (14), diarrhea (10), lower back pain (8), sweating (5), sputum (4), constipation (3), weight loss (3), tremor (2), palpitation (2), asymptomatic (160)
Lung (n = 250)	Cough (132), chest pain (99), dyspnea (45), fever (34), sputum (34), hemoptysis (21), hydatoptysis (18), nausea and vomiting (14), weakness and anorexia (11), abdominal pain (9), weight loss (3), asymptomatic (22)
Spleen (n = 21)	Abdominal pain (16), nausea and vomiting (3), swelling in the abdominal wall (3), lower back pain (2), fever (1), weakness and anorexia (1), asymptomatic (1)
Muscle (n = 14)	A painful palpable mass (12), a painless palpable mass (2)
Kidney (n = 11)	Abdominal pain (8), lower back pain (2), chest pain (1), weight loss (1), weakness and anorexia (1)
Bone (n = 10)	A painful palpable mass (5), chest pain (3), weakness in the extremities (3), back pain (2), lower back pain (1)
Intracranial (n = 8)	Headache (6), weakness in the extremities (4), dizziness (1), nausea and vomiting (1), speech impairment (1), tremor (1)
Cardiac (n = 6)	Chest pain (5), dyspnea (1), palpitation (1), fever (2), weight loss (1), asymptomatic (1)
Mediastinum (n = 3)	Dyspnea (2), chest pain (1), bloating sensation (1), asymptomatic (1)
Pelvic space (n = 3)	Abdominal pain (3)
Subcutan (n = 3)	Swelling under the skin (1), a painful palpable mass (1), asymptomatic (1)
Omentum (n = 3)	Abdominal pain (3), nausea and vomiting (1), constipation (1)
Diaphragma (n = 1)	Chest pain (1)
Pancreas (n = 1)	Abdominal pain (1)

**Table 2.** Management of Hydatid Cysts

Localization	Management	N	%
Liver (n = 969)	Surgery	363	37.5
	Albendazole + watch and wait	350	36.1
	Percutaneous therapy	236	24.3
	Surgery and percutaneous therapy	20	2.1
Lung (n = 250)	Surgery	241	96.4
	Only albendazole	9	3.6
Spleen (n = 21)	Surgery	9	42.9
	Albendazole + watch and wait	7	33.3
	PAIR	5	23.8
Muscle (n = 14)	Surgery	11	78.6
	PAIR	3	21.4
Kidney (n = 11)	Surgery	9	81.8
	Only albendazole	2	18.2
Bone (n = 10)	Surgery	8	80
	Only albendazole	2	20
Intracranial (n = 8)	Surgery	8	100
	Only albendazole	1	16.7
Mediastinum (n = 3)	Surgery	3	100
Pelvic space (n = 3)	Surgery	3	100
Subcutan (n = 3)	Surgery	3	100
Omentum (n = 3)	Surgery	3	100
Diaphragma (n = 1)	Surgery	1	100
Pancreas (n = 1)	Surgery	1	100

PAIR, puncture-aspiration-injection-reaspiration.
